# Statistical Analysis of Metagenomics Data

**DOI:** 10.5808/GI.2019.17.1.e6

**Published:** 2019-03-31

**Authors:** M. Luz Calle

**Affiliations:** Biosciences Department, Faculty of Science and Technology, University of Vic – Central University of Catalonia, Vic 08500, Spain

**Keywords:** biomarkers, DNA sequence analysis, metagenome, microbiota, statistical models

## Abstract

Understanding the role of the microbiome in human health and how it can be modulated is becoming increasingly relevant for preventive medicine and for the medical management of chronic diseases. The development of high-throughput sequencing technologies has boosted microbiome research through the study of microbial genomes and allowing a more precise quantification of microbiome abundances and function. Microbiome data analysis is challenging because it involves high-dimensional structured multivariate sparse data and because of its compositional nature. In this review we outline some of the procedures that are most commonly used for microbiome analysis and that are implemented in R packages. We place particular emphasis on the compositional structure of microbiome data. We describe the principles of compositional data analysis and distinguish between standard methods and those that fit into compositional data analysis.

## Introduction

The study of the human microbiome and its role in human health is an active area of research. The human microbiome is involved in a large number of essential functions, like food digestion and modulation of the immune system, and alterations in microbiome composition may have important effects on human health. Many diseases have already been found to be associated with changes in the human microbiome. Different studies have shown that obesity is indeed partly determined by the composition of our gut microbiome. Chronic inflammatory skin conditions such as psoriasis, atopic dermatitis, acne and chronic skin ulcers have been associated to cutaneous microbiome changes. The colonic microbiota is suspected to be involved in the development of colorectal cancers. Inflammatory bowel diseases have long been associated to interactions between microbes and the host since the microbiome is essential for the activation of host immune responses. Microbial diversity is significantly diminished in Crohn disease. Early childhood antibiotic exposure has been associated with significantly increased risk for Crohn disease [1,2]. Understanding the role of the microbiome in human health and how it can be modulated is becoming increasingly relevant for preventive medicine and for the medical management of chronic diseases.

**Fig. 1.**
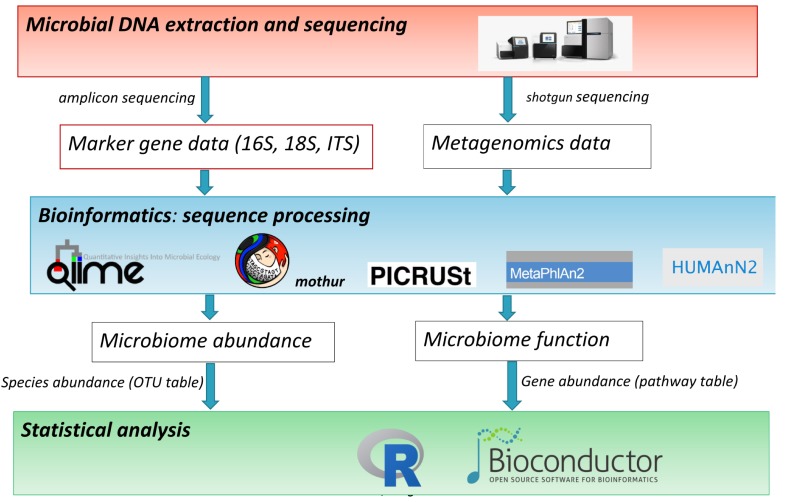
Main steps of a microbiome study: (1) microbial DNA extraction and sequencing, (2) bioinformatics sequence processing, and (3) statistical analysis.

The terms microbiome and microbiota are used indistinctly to describe the community of microorganisms that live in a given environment. High-throughput DNA sequencing technologies have powered microbiome research by enabling the study of the genomes of all microorganisms of a given environment and a more precise quantification of microbiome abundances and function. Fig. 1 summarizes the main steps of a microbiome study: (1) microbial DNA extraction and sequencing according to two main approaches, amplicon sequencing and shotgun sequencing; (2) bioinformatics sequence processing; and (3) statistical analysis.

Amplicon sequencing relies on sequencing a phylogenetic marker gene after polymerase chain reaction (PCR) amplification. For bacteria and archaea, the marker gene is the 16S ribosomal RNA gene that encodes the RNA component of the small ribosomal subunit. The 16S rRNA gene contains both highly conserved areas and hypervariable sites, denoted as V1–V9. The conserved regions can be targeted with PCR primers while the hypervariable regions are specific to each microbial species and make possible to distinguish the different microbes. The V1–V3 and V4 regions are most commonly targeted. PCR amplification creates thousands to millions of copies (amplicons) of the DNA target region. PCR amplicons are then sequenced using high-throughput sequencing platforms and multiple nucleotide sequences, also known as reads, are obtained [3].

There are a number of bioinformatic pipelines available for processing microbiome 16S sequence data, the two most popular for amplicon sequencing are mothur [4] and QIIME [5]. Both pipelines are user-friendly and produce similar results. The bioinformatics pipeline consists of five main steps: Preprocessing and quality control filtering, operational taxonomic unit (OTU) binning, taxonomy assignment, construction of the abundance table and phylogenetic analysis.

Preprocessing and quality control filtering consists on first assign the sequences to samples (demultiplexing) and then sequences are quality filtered to remove too short sequences, too many ambiguous base pairs and chimeras. OTU binning is the process of clustering similar DNA sequences into OTUs, that is, groups of DNA sequences with at least 97% similarity. The different sequences assigned to an OTU are represented by a consensus sequence determined by the most common nucleotide at each position. Taxonomy assignment is then obtained by comparing OTU consensus sequences to microbial 16S rRNA reference databases such as GreenGenes (http://greengenes.second.genome.com), SILVA (http://www.arb-silva.de), or RDP (http://rdp.cme.msu.edu). Taxonomy assignment provides the available annotation of each OTU to the different taxonomy levels (domain, kingdom, phylum, class, order, family, genus, and species). In practice, many OTUs are not completely annotated, especially for low taxonomy levels. Next, an OTU abundance table is built where each entry in the table corresponds to the number of sequences (reads) observed for each sample corresponding to each OTU. OTU tables may be extremely sparse with many OTUs only observed in a few samples. In this case it is convenient to agglomerate OTUs at broader taxonomic groups or taxa. The last step of the bioinformatics pipeline is phylogenetic analysis. Phylogenetic trees can be used to obtain phylogenetic distances between samples.

Shotgun metagenomics sequencing involves sequencing the total microbial DNA of a sample, instead of just a particular marker gene. With this technique, we can infer the relative abundance of every microbial gene and quantify specific metabolic pathways to predict the potential functionality of the entire community. This is achieved by mapping the obtained sequences against a database such as Kyoto Encyclopedia of Genes and Genomes (KEGG; http://www.genome.jp/kegg/pathway.html). A gene pathway table resulting from this type of functional study provides the number of sequences associated to a particular function for each sample. HumanN2 [6] and MetaPhlAn 2 [7] are two bioinformatics pipelines for metagenomics analysis.

From a statistical point of view, the output of both microbiome approaches, amplicon and shotgun sequencing, is similar: an abundance table of counts representing the number of sequences per sample for a specific taxon or the number of sequences matching a specific gene function. In this paper we illustrate the methodologies with data from 16S rRNA amplicon sequencing but most approaches also apply for microbiome shotgun metagenomics.

There are many reasons why the analysis of microbiome data is so challenging. On one hand, we face the usual challenges of count data analysis, i.e., skewed distribution, zero inflation and over-dispersion. Because of the experimental process and quality control filtering, microbiome data is very noisy and the total number of counts per sample is highly variable, which requires some normalization prior to the analysis so that the microbiome abundances among the different samples are comparable. Abundance tables are usually sparse since many species are infrequent. There is much redundant information because of co-abundance of many species. Moreover, the total number of counts per sample is constrained by the maximum number of sequence reads that the DNA sequencer can provide. This total count constraint induces strong dependencies among the abundances of the different taxa characterizing the compositional structure of microbiome data. Ignoring the compositionality of microbiome data may yield spurious results. In section 2, we describe the main principles of compositional data analysis.

The statistical analysis of microbiome abundance data usually starts with the normalization of the data followed by an exploratory study of the microbiome composition for the identification of possible data structures. The exploratory part consists of the analysis of diversity measures and their visualization through ordination plots, a term used in ecology to refer to several multivariate techniques for visualization of species abundance in a low-dimensional space. Subsequently, an inference analysis is performed where microbiome composition is tested for association with a variable of interest; this is known as differential abundance testing when the outcome of interest is dichotomous (i.e., disease status). These association tests can be multivariate, when the interest is to assess for global differences in microbial composition between sample groups, or univariate, with the aim of identifying which taxa are differentially abundant between sample groups. However, as we discuss later, univariate approaches for microbiome analysis are questionable and their results should be regarded with caution.

In sections 3 and 4, we describe the procedures that are commonly performed in a microbiome statistical analysis: normalization, diversity analysis, ordination and differential abundance testing, both, multivariate and univariate. This is not intended to be an exhaustive or systematic review of all the available methods. We outline some of the most widely used techniques for microbiome analysis, especially those that are implemented in R packages. We distinguish between standard methods and those that fit into compositional data analysis.

## Microbiome Compositional Data

Microbiome data is compositional because the information that abundance tables contain is relative. In a microbiome abundance table, the total number of counts per sample is highly variable and constrained by the maximum number of DNA reads that the sequencer can provide. This total count constraint induces strong dependencies among the abundances of the different taxa; an increase in the abundance of one taxon implies the decrease of the observed number of counts for some of the other taxa so that the total number of counts does not exceed the specified sequencing depth. Moreover, observed raw abundances and the total number of reads per sample are non-informative since they represent only a fraction or random sample of the original DNA content in the environment. These characteristics of microbiome abundance data clearly fall into the notion of compositional data.

Compositional data are defined as a vector of strictly positive real numbers

$$x\;=\;(x_{1},\;\ldots \;,\;x_{k}\;);\;x_{𝑖}\;>\;0\;,\;𝑖\;\in \;\{1,\;\ldots \;,\;k\}$$

with a constraint or non-informative total sum. The elements of a composition are called components or parts. In a composition the value of each component is not informative by itself and the relevant information is contained in the ratios between the components or parts [8]. Except for the fact that microbiome abundance tables contain many zeros, microbiome data fit the definition of compositional data and, as already acknowledged by many authors [9,10], their analysis requires the use of a proper mathematical theory [11]. Aitchison introduced the log-ratio approach and laid the foundations of Compositional Data Analysis (CoDA).

Mathematically, the assertion that the relevant information is contained in the ratios between the components implies that two proportional compositions are equally informative and this induces equivalence classes of vectors carrying the same information. Two vectors are compositionally equivalent if they are proportional. Each equivalence class has a representative in the unit simplex defined as:

$$S{\;}^{k\;}=\;\{\;x\;=\;(x_{1},\;\ldots \;,\;x_{k}\;),\;x_{𝑖}>0\;,\;\sum_{i=1}^{k}x_{1}=1\}$$

The simplex is thus the sample space of compositional data. In microbiome analysis, for example, both the raw counts and their transformation into relative abundances or proportions belong to the same equivalence class and they carry the same relative information.

Three important conditions should be fulfilled for a proper analysis of compositions: permutation invariance, scale invariance and sub-compositional coherence [11]. Permutation invariance states that a change in the order of the parts in the composition should not affect the results. Scale invariance establishes that any function used for the analysis of compositional data must be invariant for any element of the same compositionally equivalent class. Sub-compositional coherence requires that the results obtained when a subset of components is analyzed is coherent with the results for the whole composition. In the context of microbiome analysis this principle is important because we usually work with sub-compositions obtained after filtering out the most low-abundant taxa. Ignoring the compositional nature of microbiome data can result in spurious correlations and sub-compositional incoherencies.

Aitchison [11] put the basis of CoDA by introducing what is now called the Aitchison's log-ratio approach. The log-ratio analysis was introduced in order to meet the principle of scale invariance; as stated by Aitchison [11], “any meaningful (scale-invariant) function of a composition can be expressed in terms of ratios of its components.” Because the logarithmic transformation makes ratios mathematically more tractable, the simplest invariant function is given by the log-ratio between two components, that is:

$$𝑓(x)\;=\;\log\;(\;\;\frac{x_{𝑖}}{x_{𝑗}\;})\;,\;𝑖,𝑗\;\in \;\{1,\;\ldots \;,\;k\}$$

The generalization of a log-ratio is a log-contrast function defined as a linear combination of logarithms of the components with the restriction that the sum of the coefficients is equal to 0:

$$𝑓(x)\;=\sum_{i=1}^{k}a_{i}\log(x_{i});so\;that\;\sum_{i=1}^{k}a_{i}=0$$

Log-contrast functions are suitable for CoDA because they are scale invariant.

As an alternative to working in the simplex, several data transformations have been proposed that transform compositional data to the real space where classical statistical analysis can be applied. All of them are based on log-ratios between components.

The additive log-ratio transformation (alr) is the first proposal introduced by Aitchison [11]. Taking one part as the reference, for instance x_k_, the alr transformation is defined as:

$$𝑎𝑙𝑟(x_{1},\;\ldots \;,\;x_{k}\;)\;=\;\left(\log\left(\frac{x_{1}}{x_{k}}\right),...,\log\left(\frac{x_{k-1}}{x_{k}}\right)\right)$$

Aitchison also defined the centered log-ratio transformation (clr) to treat the parts symmetrically. The clr transformation is given by:

$$𝑐𝑙𝑟(x_{1},\;\ldots \;,\;x_{k}\;)\;=\left(\log\left(\frac{x_{1}}{g{(}_{x})}\right),...,\log\left(\frac{xk}{g(x)}\right)\right)$$

where g(x)=${\left(\prod x_{i}\right)}^{1/k}$ is the geometric mean of the composition. One characteristic of the clr transformation is that the transformed components are restricted to have a sum equal to zero and this implies that some common statistical analyses cannot be applied after the clr transformation because of a singular covariance matrix.

The third alternative is the isometric log-ratio transformation (ilr) and consists in the representation of a composition given a particular orthonormal basis in the simplex. It overcomes the problem of the singular covariance matrix present in the clr-transformation. For a detailed description see Egozcue et al. [12].

## Exploratory Analysis of Microbiome Data

The main element of a microbiome study is the microbiome abundance table, a matrix of counts, X, with n rows (samples) and k columns (taxa) where each entry *x_ij_* provides the number of sequences (reads) corresponding to taxon *j* in sample *i*. Sometimes abundance tables are transposed, rows are taxa and columns are samples. Apart from the abundance table, other elements that may be available for microbiome analysis are the sample data, the taxonomy table, and the phylogenetic tree. Several R and Bioconductor packages, such as phyloseq, are designed to facilitate the integration of all these elements in a microbiome analysis [13].

### Normalization

The large variability of the total counts per sample prevents meaningful comparisons of raw abundances between individuals. This is usually addressed through normalization of raw counts before the analysis. The most simple and frequently used normalization is the computation of relative abundances by dividing the raw abundances by the total number of counts per sample. Another popular normalization approach is rarefaction, which consists on subsampling the same number of reads for each sample so that all samples have the same number of total counts. Rarefaction is not recommended because it entails the loss of important information [14]. More sophisticated normalization techniques are implemented in some R packages, such as, DESeq [15] or edgeR [16], initially developed for RNA-seq analysis, that are also used for microbiome differential abundance testing. See Weiss et al. [17] for a comparison and discussion on the performance of different normalization methods for microbiome analysis.

CoDA techniques do not require the normalization step because the log-ratio approach involves working with ratios between components and this cancels the effect of the total counts per sample. Instead, CoDA methods entail the imputation of zeros. Microbiome abundance tables are sparse, they contain many zeros, and this should be properly addressed before compositional data methods can be applied. The simplest approach is to replace zeros by a small pseudo-count or to add a small constant to all the elements of the abundance matrix. As an alternative, Martín-Fernández et al. [18] propose the Bayesian-Multiplicative treatment, a zero replacement involving Bayesian inference and a modification of the non-zero values so that the original ratios between the non-zero components are preserved.

### Diversity analysis

The diversity of the microbiome is an important indicator of the good or bad conditions of the ecosystem, with larger microbiome diversity being usually associated to better health status. Microbiome diversity can be assessed through multiple ecological indices that can be divided into two kind of measures, alpha and beta diversity. Alpha diversity measures the variability of species within a sample while beta diversity accounts for the differences in composition between samples. The R package vegan provides a large set of diversity measures [19].

#### Alpha diversity: within sample diversity

The most important measure of alpha diversity is richness, defined as the number of different species present in an environment. Richness is estimated by the observed richness, *R_obs_*, the number of different species observed in the sample. The observed richness tends to underestimate the real richness in the environment, where the less frequent species are likely to be undetected. There are different indices that adjust for this and try to estimate the hidden part that has not been detected. One of the most extended richness measure is Chao1 index defined as

$${𝑅}_{𝐶ℎ𝑎𝑜1}\;=\;{𝑅}_{𝑜𝑏𝑠}\;+\frac{{𝑓}_{1}({𝑓}_{1}\;-\;1)}{2({𝑓}_{2}\;+\;1)}$$

where *f_1_* is the number of species observed only once and *f_2_* is the number of species observed twice.

Another important indicator of alpha diversity is evenness, which measures the homogeneity in abundance of the different species in a sample. A commonly used measure of evenness is the Shannon index defined as

$${𝑅}_{Sℎ𝑎𝑛𝑛𝑜𝑛}\;=\;-\sum_{i=1}^{k}p_{i}\log\left(p_{i}\right)$$

where *p_i_* represents the relative abundances of the i-th taxon.

#### Beta diversity: between samples diversity

Beta diversity measures the differences in microbiome composition between samples. There is a wide range of ecological distances or dissimilarities for measuring how close are two microbial compositions. The most commonly used are Bray-Curtis, UniFrac and weighted UniFrac distances. We also define the Aitchison distance which is a proper distance for compositional data.

Let *p*_1_= (*p_11_,…,p_1k_* ) and *p*_2_= (*p_21_,…,p_2k_* ) denote the microbiome relative abundance of two different samples.

Bray-Curtis is defined as follows:

$$d_{BC}\left(p_{1},p_{2}\right)=\frac{\sum_{i=1}^{k}\left|p_{1i}-p_{2i}\right|}{\sum_{i=1}^{k}\left(p_{1i}+p_{2i}\right)}$$

UniFrac family of distances [20] consider the phylogenetic tree that represents the evolutionary relationships among the different taxa. The phylogenetic tree can be obtained from the bioinformatic pipelines, such as mothur and QIIME. For a tree with r branches, let *b* = *(b1,…,br*) represent the length of the different branches in the phylogenetic tree, and *q_1_= (q_11_,… ,q_1r_*), and *q2= (q_21_,… ,q_2r_*) the relative abundances associated to each branch for the first and the second sample, respectively.

The unweighted UniFrac distance measures the relative length of those branches that lead exclusively to species present in only one of the two samples with respect to the total length of all branches in the tree:

$$d_{U}{(}_{b,q1,q2})=\frac{\sum_{i=1}^{r}bi\left|I\left(q_{1i}>0\right)-I\left(q_{2i}>0\right)\right|}{\sum_{i=1}^{r}b_{i}I\left(q_{1i}+q_{2i}>0\right)}$$

The unweighted UniFrac distance only takes into account the presence or absence of the taxa but Lozupone et al. [20] also introduced the weighted UniFrac distance that includes information on the relative abundance of each taxa and is defined as follows:

$$d_{W}{(}_{b,q1,q2})=\frac{\sum_{i=1}^{r}b_{i}\left|q_{1i}-q_{2i}\right|}{\sum_{i=1}^{r}b_{i}\left(q_{1i}+q_{2i}\right)I\left(q_{1i}+q_{2i}>0\right)}$$

For a proper CoDA analysis, a distance must be subcompositionally dominant, which means that the distance between two points in a multi-dimensional space should always be larger than their distance when projected in a lower dimensional space (sub-composition). Most commonly used distances in microbiome analysis, such as, the Bray-Curtis and the weighted and unweighted UniFrac distances are not sub-compositionally dominant, and this may induce sub-compositionally incoherencies that question the reliability of the results of any distance-based analysis [8,11,21].

The Aitchison distance is a sub-compositionally coherent distance defined as the Euclidean distance after the clr-transformation of the compositions. Given two compositions *x_1_* and *x_2_*, the Aitchison distance is given by

$${𝑑}_{𝐴}(x_{1},\;x_{2}\;)\;=\;{𝑑}_{𝐸}\;(𝑐𝑙𝑟(x_{1}),\;𝑐𝑙𝑟(x_{2}))$$

where *d_E_* denotes Euclidean distance.

### Ordination

The goal of ordination plots is the visualization of beta diversity for identification of possible data structures. The multidimensional data is represented into a reduced number of orthogonal axes while keeping the main trends of the data and preserving the distances among samples as much as possible. Most commonly used ordination methods for microbiome data are principal coordinates analysis (PCoA), also known as multidimensional scaling, and non-metric multidimensional scaling (NMDS) [22,23].

PCoA an extension of Principal Components Analysis (PCA). Given a distance or dissimilarity matrix, *D*, PCoA performs eigenvalue decomposition of *D_c_*'*D_c_* where *D_c_* is the centered distance matrix. When *D* is the Euclidean distance, PCoA results exactly the same as PCA. Care must be taken with PCoA if the selected distance is not metric, because some eigenvalues may be negative and then, the graphical representation will not perform properly.

In order to avoid this problem NMDS is more commonly used. Also based on a distance matrix *D*, NMDS maximizes the rank-based correlation between the original distances and the distances between samples in the new reduced ordination space. The procedure starts with a random configuration and the optimal representation is obtained following an iterative procedure that at each steps improves the rank correlation.

Ordination plots can be obtained with the R and Bioconductor packages vegan and phyloseq, among others [13,19]. Alternatively, a CoDA ordination approach can be followed by performing PCA after the clr or ilr transformation as implemented by Le Cao et al. [24] in the context of the multivariate statistical framework mixMC.

## Microbiome Statistical Inference

### Multivariate differential abundance testing

Multivariate differential abundance testing refers to a global test of differences in microbial composition between two or more groups of samples. We can distinguish between distance-based or model-based approaches.

Permutational Multivariate Analysis of Variance Using Distance Matrices, PERMANOVA [25], is perhaps the most widely used distance-based method for multivariate community analysis. The null hypothesis of no differences in composition among groups is formulated by the condition that the different groups of samples have the same center of masses. Implemented in the function “adonis” of the vegan R package, it consists of a multivariate ANOVA based on dissimilarities. The variability within groups is compared against the variability between groups with the usual ANOVA F statistic, but partition of sums-of-squares is applied directly to dissimilarities. Significance is evaluated through permutations to generate a distribution of the pseudo F statistic under the null.

A related and popular distance-based approach is the analysis of similarities [26], implemented in the function “anosim” of the vegan R package.

An interesting model-based approach for multivariate microbiome analysis is Kernel machine regression (KMR), that extends PERMANOVA to a regression framework [27]. KMR is a semi-parametric regression model that includes a nonparametric component. The model can be expressed as a semiparametric linear regression model when the response variable is continuous

$${𝑦}_{𝑖}\;=\;{𝛽}_{0}\;+\;𝛽\;\prime {𝑍}_{𝑖}\;+\;ℎ({𝑋}_{𝑖}\;)\;+\;{𝜖}_{i}$$

or as a semiparametric logistic regression model for a dichotomous response variable

$$𝑙𝑜𝑔𝑖𝑡({𝑦}_{𝑖}\;)\;=\;{𝛽}_{0}\;+\;𝛽\;\prime {𝑍}_{𝑖}\;+\;ℎ({𝑋}_{𝑖})\;+\;{𝜖}_{𝑖\;}$$

In the context of microbiome analysis, X is the microbiome abundance matrix and the non-parametric component *h*(X) measures the relationship between microbiome composition and the outcome. This association can be tested according to the following hypothesis:

$${𝐻}_{0}:\;ℎ(𝑋)\;=\;0\;\;𝑣𝑠\;\;{𝐻}_{1}:\;ℎ(𝑋)\;\neq \;0$$

The nonparametric component is related to a Kernel matrix that is a transformation of the distance matrix *D* of pairwise distances between individuals. KMR is implemented for microbiome analysis in the R package MiRKAT [28]. KMR can be adapted to CoDA by using a subcompositionally dominant function, such as, the Aitchison distance. Rivera-Pinto [29] has implemented this adaptation in the R package MiRKAT-CoDA. The algorithm also includes a weighted version that allows the identification of the taxa that are more relevant for the joint association.

Among the different model-based methods for microbiome differential abundance testing we highlight the work by La Rosa et al. [30] that consider the Dirichlet-Multinomial distribution for hypothesis testing, power and sample size calculations. The proposed methods are implemented in the R package HMP.

Le Cao et al. [24] propose the multivariate statistical framework mixMC where they perform sparse partial least squares discriminant analysis (sPLS-DA), implemented in the R package mixOmics [31]. In order to acknowledge the compositional structure of microbiome data, they apply sPLS-DA after the clr transformation. PLS-DA maximizes the covariance between linear combinations of the taxa and the response variable. The sparse version of PLS-DA uses Lasso penalized regression [32] and thus, it performs variable selection that enables the identification of the taxa that are most associated with the outcome.

### Univariate differential abundance testing

When significant global differences in microbiome composition are detected between groups of samples, a natural question arises: which particular taxa are responsible of that global difference? A common strategy to answer this question is to test every taxa separately for association with the response variable. When the response variable is dichotomous this is known as univariate differential abundance testing.

Below we describe both, classical and CoDA approaches for univariate differential abundance testing. However, we advise that classical univariate approaches are notably affected by the compositional structure of microbiome data and their results, with large false discovery rates, might be questioned [17,33].

Nonparametric tests, like the Wilcoxon rank-sum test or the Kruskal-Wallis test, can be applied. However, more powerful parametric approaches are available, such as the Bioconductor packages edgeR [16] and DESeq2 [34], initially proposed for transcriptomics analysis (RNA-Seq data). Both fit a generalized linear model and assume that read counts follow a Negative Binomial distribution. The NB distribution extends the Poisson distribution by allowing the variance to be different from the mean. edgeR and DESeq2 mainly differ in the way they normalize the data. DESeq2 uses size factors that account for differences in sequencing depth between samples and shrinkage for large variances correction. edgeR can be implemented with different normalization methods but the most recommended is TMM, the trimmed mean of M-values normalization method, that indirectly attempts to overcome the problem of compositional DNA sequencing data ("the proportion of reads attributed to a given gene in a library depends on the expression properties of the whole sample rather than just the expression level of that gene") [16].

Two CoDA methods that explicitly accounts for the compositional nature of microbiome data are ANCOM [35] and ALDEx2 [36]. In ANCOM, the log-ratio of all pairs of variables is tested for differences in means. The number of significant results involving each variable is used to determine its significance. The ALDEx2 algorithm uses a Dirichlet-multinomial model to infer the multivariate abundance distribution from counts. After clr transformation it performs the Wilcoxon rank test (two groups) or Kruskal-Wallis tests (more than two groups).

### Microbial signatures

Recently, Rivera-Pinto et al. [37] have proposed a new CoDA approach for microbiome analysis that is aimed to the identification of microbial signatures, groups of microbial taxa that are predictive of a phenotype of interest. The identification of microbial signatures involves both modeling and variable selection: modeling the response variable and identifying the smallest number of taxa with the highest prediction or classification accuracy. In order to fulfill the principles of CoDA, instead of analyzing individual abundances, we analyze the relative abundances between two groups of taxa, also referred as the abundance balance between the two groups, a concept that is formally defined as follows:

Let *x =(x_1_,x_2_,...,x_k_*) be the microbial composition of *k* taxa and, among these *k* taxa, let's consider two disjoint subgroups of taxa, group *A* and group *B*, with composition abundances denoted by *x_A_* and *x_B_*, each group with *k_A_* and *k_B_* different taxa and indexed by *I_A_* and *I_B_*, respectively. The abundance balance between *A* and *B*, denoted by *B*(*A*,*B*), is defined as the log-ratio between the geometric mean abundances of the two groups of taxa as follows:

$$B(𝐴,\;𝐵)\;=\;𝐶\;∙\;𝑙𝑜𝑔\frac{{\left({\prod_{𝑖\in 𝐼}}_{𝐴}\;x_{i}\right)}^{\frac{1}{k_{A}}}}{{\left({\prod_{𝑗\in 𝐼}}_{𝐵}\;x_{i}\right)}^{\frac{1}{k_{B}}}}$$

where C is a normalization constant. The larger the values of balance *B*(*A*,*B*), the more abundant is group A with respect to group B. Positive values of *B*(*A*,*B*) arise when group A is more abundant than group B while negative values of *B*(*A*,*B*) correspond to larger abundance of group B relative to group A abundance. A value of *B*(*A*,*B*)=0 correspond to a perfect balance between the abundances of both groups of taxa.

The goal of the proposed algorithm is the identification of the two groups of microbial taxa, group *A* and group *B*, whose abundance balance *B*(*A*,*B*) is most associated with an outcome of interest Y. For instance, for a binary outcome *Y* corresponding to disease status (*Y*=1 for diesased and *Y*=0 for not diseased), if we are able to identify the two groups of taxa *A* and *B* whose balance is associated with *Y* we may use *B*(*A*,*B*), the relative abundance between groups *A* and *B*, as a microbial signature of disease risk. If large values of *B*(*A*,*B*) are associated with *Y*=1, we will infer that a person with larger relative abundances of group A with respect to group B will have higher risk of disease than other people with lower relative abundances between *A* and *B*.

The algorithm for the selection of microbial balances is implemented in the R package selbal. It starts with a first thorough search of the two taxa whose balance, or log-ratio, is most associated with the response variable. Once the first two-taxon balance is selected, the algorithm performs a forward selection process where, at each step, a new taxon is added to the existing balance such that the specified optimization criterion is improved (area under the receiver operating characteristic or mean squared error). The algorithm stops when there is no additional variable that improves the current optimization parameter or when the maximum number of components to be included in the balance is achieved. This number is established with a cross-validation procedure, which is also used to explore the robustness of the identified balance.

## Discussion

In this work we present some of the techniques that are most commonly used for microbiome analysis. We place a particular emphasis on those methods that preserve the principles of compositional data analysis.

Classical methods that ignore the compositional nature of microbiome data can result in spurious correlations and sub-compositional incoherencies. This is especially relevant for classical univariate test where the strong dependencies between microbial abundances results in an important increase of type I error. Simulation studies show that the false discovery rate increases as the true-positive fold change increases and that it can achieve unacceptable extremely large values [17,33]. Moreover, from a biological point of view univariate approaches are questionable because they ignore that the microbiome is an ecosystem with complex interactions between its members and with the environment.

There is an increasing awareness of the need of using proper CoDA methods for microbiome analysis [10,38]. In this work we make clear that proper CoDA methods are available for all steps of a microbiome statistical analysis: normalization, diversity analysis, ordination and differential abundance testing, both, multivariate and univariate.

Normalization is not required and only zero imputation is needed. Diversity analysis and ordination can be performed after clr or ilr transformations, for instance, Aitchison distance and PCA. CoDA adapted Kernel machine regression can be used for multivariate differential abundance testing. Univariate approaches are not recommended. Penalized multivariate regression, such as sPLS-DA, is an alternative for the identification of the taxa that are most associated with the outcome. The algorithm selbal for the selection of microbial signatures is also an alternative to univariate selection of taxa when the main interest is prediction.

Even so, more research is still needed to fully understand the performance and limitations of the current available CoDA methods for microbiome analysis that will probably lead to their improvement or to the proposal of new approaches.
